# Xanthine Oxidase Inhibition by Febuxostat Attenuates Experimental Atherosclerosis in Mice

**DOI:** 10.1038/srep04554

**Published:** 2014-04-01

**Authors:** Johji Nomura, Nathalie Busso, Annette Ives, Chieko Matsui, Syunsuke Tsujimoto, Takashi Shirakura, Mizuho Tamura, Tsunefumi Kobayashi, Alexander So, Yoshihiro Yamanaka

**Affiliations:** 1 Pharmaceutical Department Research Laboratories, Teijin Institute for Bio-Medical Research, Teijin Pharma Limited, Hino, Tokyo, Japan; 2 Service of Rheumatology, Department of l'Appareil Locomoteur, Centre Hospitalier Universitaire Vaudois, University of Lausanne, Lausanne, Switzerland

## Abstract

Atherosclerosis is a chronic inflammatory disease due to lipid deposition in the arterial wall. Multiple mechanisms participate in the inflammatory process, including oxidative stress. Xanthine oxidase (XO) is a major source of reactive oxygen species (ROS) and has been linked to the pathogenesis of atherosclerosis, but the underlying mechanisms remain unclear. Here, we show enhanced XO expression in macrophages in the atherosclerotic plaque and in aortic endothelial cells in ApoE^−/−^ mice, and that febuxostat, a highly potent XO inhibitor, suppressed plaque formation, reduced arterial ROS levels and improved endothelial dysfunction in ApoE^−/−^ mice without affecting plasma cholesterol levels. *In vitro*, febuxostat inhibited cholesterol crystal-induced ROS formation and inflammatory cytokine release in murine macrophages. These results demonstrate that in the atherosclerotic plaque, XO-mediated ROS formation is pro-inflammatory and XO-inhibition by febuxostat is a potential therapy for atherosclerosis.

Atherosclerosis is a chronic inflammatory disease of arteries secondary to lipid deposition in the vessel wall and oxidative stress participates in its pathogenesis^1^,^2^,^3^. In the early phase of atherosclerosis, oxidative stress modifies LDL to oxidized LDL that is taken up by macrophages in the intima of the vascular wall, ultimately leading to the formation of foam cells. Later, cholesterol crystals (CCs) appear, which can stimulate inflammatory responses and apoptosis in foam macrophages^4^. Oxidative stress also causes endothelial dysfunction, one of the early features of atherosclerosis^5^. Therefore, oxidative stress and inflammation are intimately linked and play essential roles in the progression of atherosclerosis, and elucidation of the underlying mechanisms may lead to novel strategies for the prevention and treatment of atherosclerosis.

Oxidative stress results from the formation of reactive oxygen species (ROS), which are generated by various pathways such as xanthine oxidoreductase (XOR), NADPH oxidase and mitochondrial respiratory enzymes^6^. XOR is a molybdopterin-containing enzyme that oxidizes hypoxanthine to xanthine and finally to uric acid. XOR exists in two interconvertible forms, xanthine oxidase (XO) and xanthine dehydrogenase (XDH). Whereas XDH uses NAD+ as an electron acceptor and produces NADH, XO mainly produces ROS such as superoxide and hydrogen peroxide by preferentially using molecular oxygen as an electron acceptor^7^. XOR, as well as its metabolite uric acid, are increased in atherosclerotic plaques compared to non-atherosclerotic carotid arteries, and CCs, XOR and uric acid are co-localized in these plaques^8^. In addition, XOR inhibitors such as tungsten and allopurinol inhibited the progression of atherosclerosis in ApoE^−/−^ mice. XOR is involved not only in the uptake of oxidized LDL into macrophages but also in the induction of cytokines by soluble ligands such as LPS and atherosclerotic serum^9^,^10^,^11^. Furthermore, ROS scavengers such as N-acetyl-L-cysteine (NAC) and apocynin inhibited plaque formation and arterial inflammation in mice^12^,^13^,^14^. These findings strongly suggest that XO plays an important role in the progression of atherosclerosis through ROS generation but definitive proof of its role in atherosclerotic inflammation and the underlying mechanisms remain to be established.

Intracellular CCs are found in diet-induced atherosclerotic plaques and are composed of oxidized LDL that has undergone endocytosis^15^,^16^. In macrophages, CCs activate the NLRP3-inflammasome resulting in IL-1β secretion^15^,^16^,^17^. Intracellular ROS production has been shown to be critical for the NLRP3-dependent secretion of IL-1β in response to monosodium urate (MSU)^18^,^19^. It is not known if CC-induced pro-inflammatory cytokine release is a XO- or ROS-dependent process.

In this study, we examined the expression of XO in experimental atherosclerosis, and studied the effects of febuxostat, a highly potent inhibitor of XOR, both *in vivo* in the atherosclerosis model and *in vitro* in CC-induced inflammation in macrophages.

## Results

### Xanthine oxidoreductase is increased in macrophages and endothelial cells in ApoE^−/−^ mice

**Figure 1:**
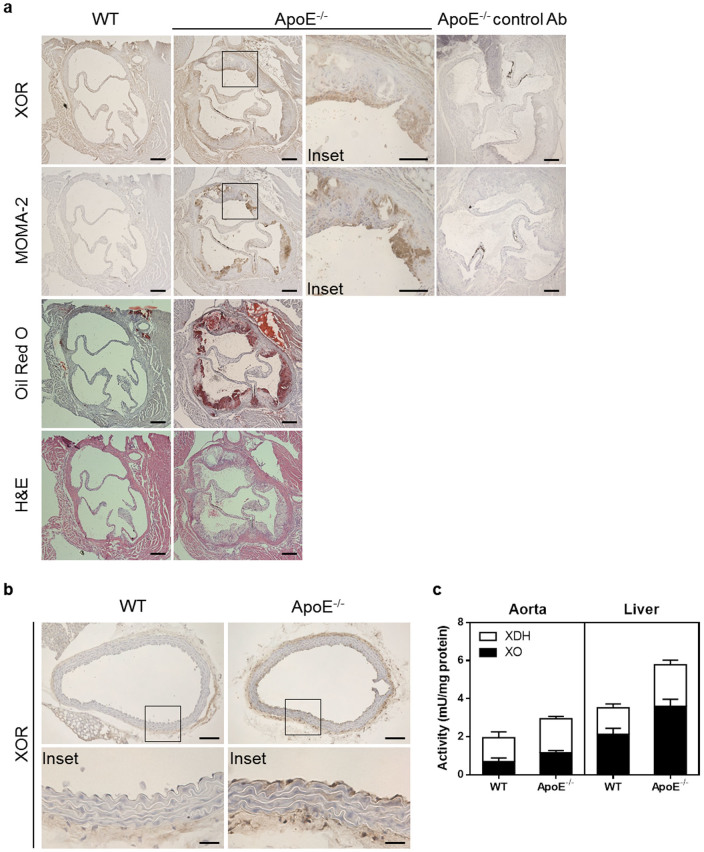
Xanthine oxidoreductase is increased in macrophages and endothelial cells in ApoE^−/−^ mice. (a), Representative photographs of XOR staining (top panels), MOMA-2 staining (middle panels), oil red O staining (middle panels), and hematoxylin-eosin staining (bottom panels) of aortic sinus. Scale bars: 200 μm. (b), Representative photographs of XOR staining of thoracic aorta. Scale bars: 100 μm (upper panels) and 25 μm (lower panels). (c), XOR activity in thoracic aorta (n = 5) and liver (n = 10) from WT and ApoE^−/−^ mice. Data are shown as mean ± SEM.

ApoE^−/−^ mice kept on a high cholesterol diet for 12 weeks showed atherosclerotic features such as the development of oil red O-positive staining and macrophage infiltration of the aortic sinus compared to non-atherosclerotic WT mice (Figure 1a). In aortic sinus from ApoE^−/−^ mice, XOR protein was abundantly expressed in MOMA-2-positive macrophages infiltrated into the plaque, whereas it was very low in non-atherosclerotic aortic sinus from WT mice. In addition, in thoracic aorta from ApoE^−/−^ mice, XOR was increased in endothelial cells and smooth muscle cells (Figure 1b). Consistent with these findings, XOR functional activity in aorta from ApoE^−/−^ mice was increased compared to WT mice. Increased XOR activity was also observed in the liver and plasma (Figure 1c and Table 1). As macrophages and endothelial cells are involved in the process of plaque formation and endothelial dysfunction, we hypothesized that XOR could play a role in both these pathogenic processes of atherosclerosis.

### Febuxostat inhibits plaque formation in ApoE^−/−^ mice

**Figure 2:**
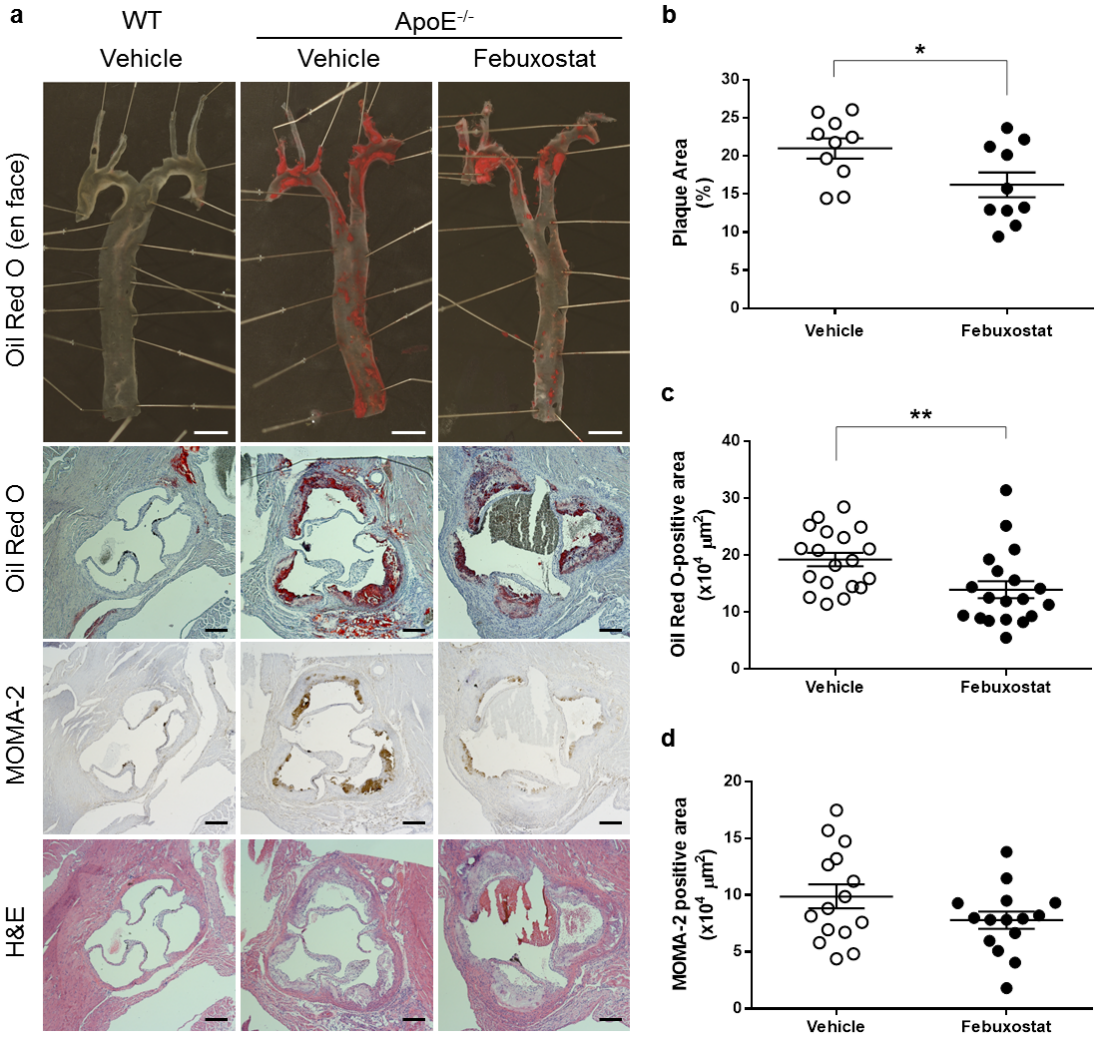
Febuxostat inhibits plaque formation in ApoE^−/−^ mice. (a), Representative photographs of *en face* oil red O staining of aorta (top panels), cross-sectional oil red O staining (middle panels), cross-sectional MOMA-2 staining (middle panels), and cross-sectional hematoxylin-eosin staining (bottom panels) of aortic sinus. Scale bars: 2 mm (top panels) and 200 μm (the other panels). (b), Quantitative analysis of *en face* oil red O-positive area of aorta from vehicle-treated (n = 10) and febuxostat-treated (n = 10) ApoE^−/−^ mice. Data are shown as mean ± SEM. *P < 0.05 versus vehicle-treated group. (c), Quantitative analysis of cross-sectional oil red O-positive area of aortic sinus from vehicle-treated (n = 19) and febuxostat-treated (n = 19) ApoE^−/−^ mice. Data are pooled from two independent experiments in which similar results were obtained, and shown as mean ± SEM. **P < 0.01 versus vehicle-treated group. (d), Quantitative analysis of cross-sectional MOMA-2-positive area of aortic sinus from vehicle-treated (n = 15) and febuxostat-treated (n = 15) ApoE^−/−^ mice. Data are pooled from two independent experiments in which similar results were obtained, and shown as mean ± SEM.

We examined whether febuxostat, a highly potent inhibitor of XOR, inhibited plaque formation and macrophage infiltration. Febuxostat (2.5 mg/kg/day) was administrated for 12 weeks in drinking water to ApoE^−/−^ mice fed a high cholesterol diet and significantly inhibited plasma XO activity at 12 weeks compared to vehicle-treated mice, showing that an effective dose of febuxostat was administrated. On the other hand, febuxostat did not change body weight, total cholesterol, triacylglycerol, NEFA, and glucose levels but increased levels of HDL were found in ApoE^−/−^ mice (Table 1). Histologically, both whole aorta and cross-sectional aortic sinus exhibited oil red O-positive lesion areas which were significantly decreased in febuxostat-treated ApoE^−/−^ mice, compared to the vehicle-treated group (Figure 2). Febuxostat did not significantly reduce MOMA-2-staining in ApoE^−/−^ mice. These results suggest that XOR inhibition by febuxostat attenuated plaque formation, but not macrophage infiltration in mice.

### Febuxostat reduces the levels of ROS in the aortic wall of atherosclerotic mice

**Figure 3:**
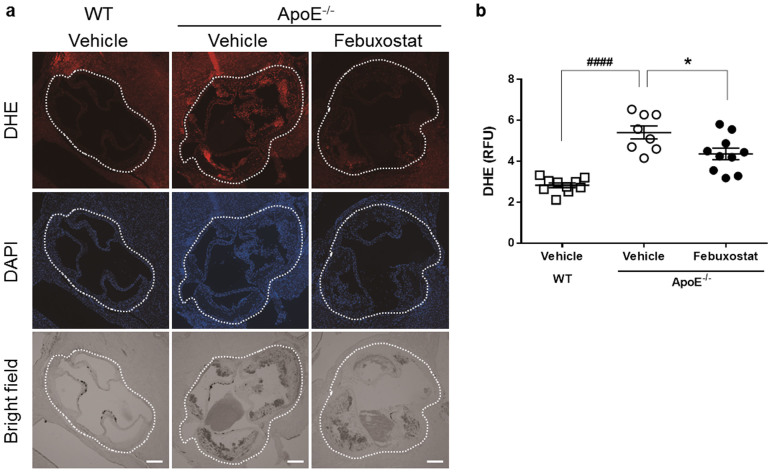
Febuxostat reduces the levels of ROS in the aortic wall of atherosclerotic mice. (a), Representative photographs of cross-sectional DHE staining (top panels), DAPI staining (middle panels), and bright field (bottom panels) of aortic sinus. Dashed lines indicate aortic sinus lesion. Scale bars: 200 μm. (b), Quantitative analysis of DHE fluorescence intensity in aortic sinus from WT (n = 10), vehicle-treated (n = 8), and febuxostat-treated (n = 10) ApoE^−/−^ mice. Data are representative of two independent experiments in which similar results were obtained, and shown as mean ± SEM. ^####^*P* < 0.0001 versus vehicle-treated WT, *P < 0.05 versus vehicle-treated ApoE^−/−^ mice.

We assessed the level of ROS expressed in the aortic sinus by DHE staining, an indicator of oxidative stress, and found that staining was significantly increased in aorta from ApoE^−/−^ mice compared to that in WT mice (Figure 3). Febuxostat significantly reduced DHE staining in ApoE^−/−^ mice. These data showed that increased XO activity in ApoE^−/−^ mice is associated with increased levels of ROS as well as the severity of atherosclerosis, and can be attenuated by febuxostat.

### Febuxostat inhibits the expression of pro-inflammatory genes in the aorta

**Figure 4:**
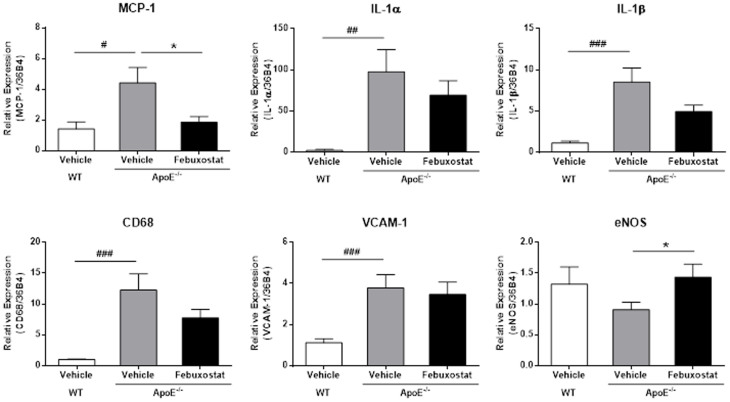
Febuxostat inhibits the expression of pro-inflammatory genes in the aorta. qRT-PCR analysis was performed using total RNA extracted from aorta of vehicle-treated WT (n = 10), vehicle-treated (n = 10), and febuxostat-treated (n = 9) ApoE^−/−^ mice. Data are pooled from two independent experiments in which similar results were obtained, and shown as mean ± SEM. ^#^*P* < 0.05, ^##^*P* < 0.01, ^###^*P* < 0.001 versus vehicle-treated WT mice, *P < 0.05 versus vehicle-treated ApoE^−/−^ mice.

Next, we examined the mRNA expression of pro-inflammatory genes in the aorta (Figure 4). Expressions of MCP-1, IL-1α and IL-1β have been reported to be associated with atherosclerosis^20^,^21^,^22^. In addition, VCAM-1 mediates the migration of macrophages into the tissue, and CD68 is a marker for macrophage lineage^23^. We found significantly increased levels of MCP-1, IL-1α, IL-1β, CD68 and VCAM-1 mRNA in ApoE^−/−^ mice. Febuxostat treatment significantly decreased MCP-1 expression, and a trend towards decreased IL-1α, IL-1β and CD68 expression levels was also observed. By contrast, VCAM-1 expression level was not affected. In addition, we examined eNOS, a mediator of endothelial-dependent relaxation and vascular protection^24^,^25^ and found that mRNA levels of eNOS in ApoE^−/−^ mice were significantly enhanced by febuxostat.

### Cholesterol crystals enhance macrophage inflammatory cytokine secretion via XO and ROS

**Figure 5:**
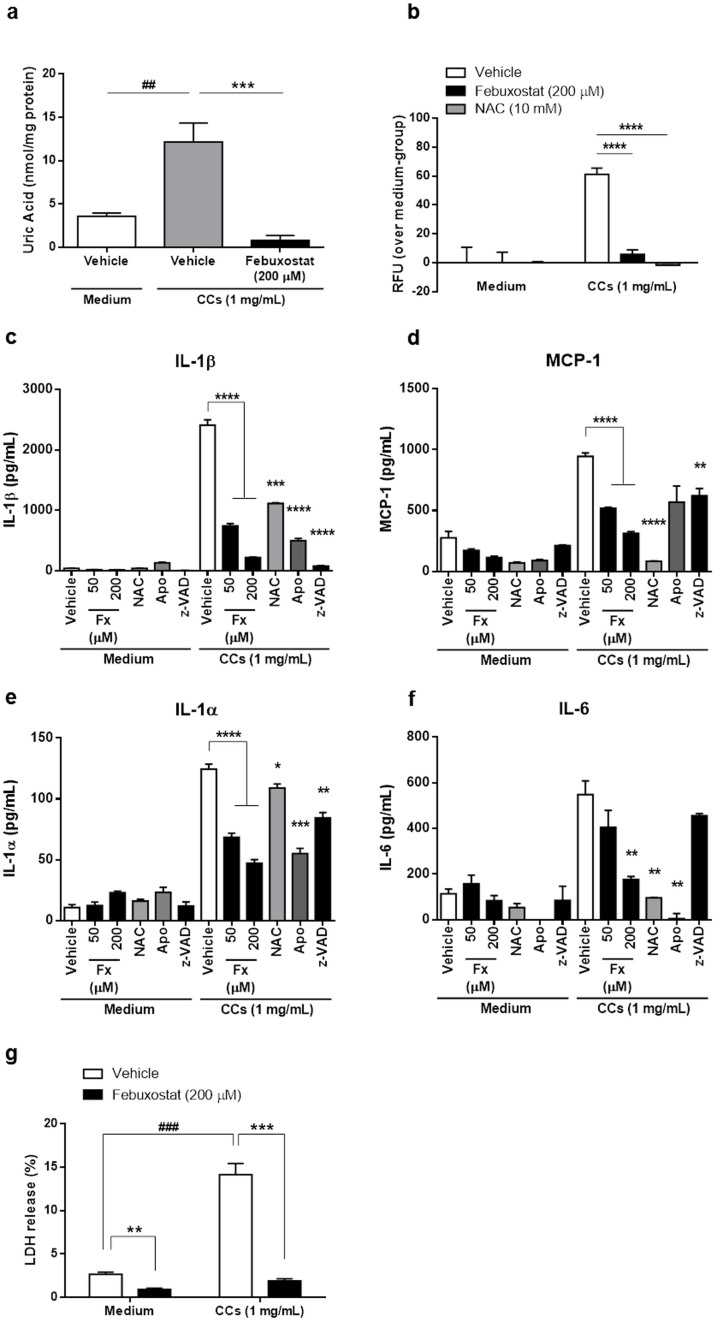
Cholesterol crystals enhance macrophage inflammatory cytokine secretion via XO and ROS. Primed BMDM were stimulated for 30 min (a) and (b) or 6 h (c–g) with CCs in the presence or absence of febuxostat. (a), The effect of CCs on intracellular uric acid level. Data are representative of three independent experiments and are shown as mean ± SEM. ^##^*P* < 0.01 versus vehicle/medium-treated, ***P < 0.001 versus vehicle/CC-treated group. (b), The effect of CCs on intracellular ROS accumulation. Data are representative of three independent experiments and are shown as mean ± SEM. ****P < 0.0001 versus vehicle/CC-treated group. (c–f), The effects of CCs on the productions of inflammatory mediators. Fx indicates febuxostat; NAC, N-acetyl-L-cysteine (50 mM); Apo, Apocynin (500 μM); z-VAD, z-VAD-fmk (10 μM), respectively. Data are representative of three independent experiments and shown as mean ± SEM. *P < 0.05, **P < 0.01, ***P < 0.001, ****P < 0.0001 versus vehicle/CC-treated group. (g), The effect of CCs on LDH release. Data are representative of three independent experiments and shown as mean ± SEM. ^###^*P* < 0.001 versus vehicle/medium-treated group, **P < 0.01, ***P < 0.001 versus vehicle-treated group.

We hypothesized that CCs stimulate macrophage pro-inflammatory cytokine release by inducing XO and ROS production. BMDM were first primed with Pam3CSK4 for activation, and then stimulated with CCs. Intracellular uric acid levels were increased by CCs, suggesting that CCs increased purine metabolism, leading to relative increase in endogenous XOR activity (Figure 5a). As expected, febuxostat completely inhibited the increased intracellular uric acid production (Figure 5a). CCs also increased intracellular ROS generation by primed macrophages (Figure 5b). This ROS induction was totally inhibited by NAC, a ROS scavenger and also by febuxostat (Figure 5b). These results indicated that CCs induced XO activity, leading to the increased ROS level. We next tested whether CCs induced pro-inflammatory cytokine release. The secretion of IL-1β by primed macrophages was inhibited by caspase inhibitor z-VAD-fmk, by ROS inhibitors as well as by febuxostat in a dose dependent fashion (Figure 5c). In addition, we found that MCP-1, IL-1α and IL-6, which all play an important role in atherosclerosis, were also induced by CC stimulation in primed macrophages (Figure 5d–f). Although caspase inhibition had a partial effect on IL-1α and MCP-1 secretion, it had no effect on IL-6 secretion. Febuxostat dose-dependently decreased the release of all these cytokines/chemokines, indicating that it inhibits the release of both caspase-1-dependent and -independent molecules (Figure 5c–f). The ROS scavengers NAC and apocynin recapitulated the inhibitory effects of febuxostat on the secretion of MCP-1, IL-1β, IL-1α and IL-6 (Figure 5c–f). In addition, it has been reported that caspase-1-dependent cell death, called pyroptosis is involved in plaque instability^26^. Febuxostat prevented lactate dehydrogenase (LDH) release induced by CCs, suggesting that febuxostat has no cytotoxicity per se and can inhibit pyroptosis induced by CCs (Figure 5g). Taken together, these results demonstrated that generation of ROS through XO activity is a crucial mechanism in CC-induced cytokine and chemokine production.

## Discussion

The roles of XO and ROS in atherosclerosis have been well documented in previous studies^9^,^10^,^12^,^13^,^14^, but the mechanisms that drive ROS generation as well as the consequences of its production are not fully understood. In this study, we present data to show that the development of atheroma in the aorta is accompanied by macrophage infiltration and co-localizes with areas of increased XO and ROS activities. Inhibition of XO and ROS production by a potent XOR inhibitor, febuxostat, attenuated not only the histological features of atherosclerosis but also the production of pro-inflammatory mediators in the aorta. Furthermore, CCs stimulate macrophages to produce inflammatory mediators implicated in atherosclerosis via a XO dependent ROS pathway. These findings suggest ROS generation by XO links CC accumulation in the atheromatous plaque to local inflammation, and its inhibition can attenuate the progression of atherosclerosis.

Atheromatous plaques are formed by crystallization of soluble oxidized LDL into CCs within macrophages^15^ and are inflammatory lesions^14^. We found that CCs are potent inducers of the secretion of inflammatory mediators such as MCP-1, IL-1α and IL-6 from macrophages and confirmed that CCs induced macrophage IL-1β secretion through caspase-1-dependent mechanisms. The importance of NLRP3/caspase-1 in atherosclerosis has been questioned. NLRP3^−/−^ApoE^−/−^ double-deficient mice have been reported to develop atherosclerosis independently of inflammasome^27^. Conversely, less atherosclerotic plaque was seen in NLRP3^−/−^ bone marrow-transplanted LDLR deficient mice or in caspase-1^−/−^ApoE^−/−^ double-deficient mice^15^,^28^. The reasons for these discrepancies are unclear, but suggest that inflammation in atherosclerosis does not always require the NLRP3-inflammasome.

Particulate DAMPs such as MSU crystals activate the NLRP3-inflammasome through ROS-dependent mechanisms^29^. Macrophages exposed to CCs showed enhanced XO activity, evidenced by increases in intracellular uric acid and ROS levels and ROS scavengers and febuxostat inhibited IL-1β production induced by CCs. We found that XO is the major source of ROS in macrophages and its generation results not only in IL-1β release, but also the secretion of IL-1α, IL-6 and MCP-1. These results were recapitulated *in vivo*, as atheromatous plaques showed increased XO activity, ROS levels and inflammatory cytokine expression. Both atherosclerosis and inflammation were attenuated when the mice were treated with febuxostat. Febuxostat itself had no effects on serum lipids in the animal model, indicating that its effects were due to XO inhibition. Taken together, we conclude that XO plays a fundamental role in atherosclerosis-related inflammation through intracellular ROS accumulation in macrophages, but not in lipid metabolism.

Both *in vivo* and *in vitro*, XO inhibition inhibited caspase-1-dependent and -independent pro-inflammatory cytokine/chemokine release. *In vitro*, CCs induced MCP-1, IL-1α and IL-6 release from macrophages. The secretion of IL-6 is caspase-1-independent, as it was not altered when z-VAD-fmk was added; whereas the induction of IL-1α and MCP-1 was partially dependent on caspase-1. *In vivo*, MCP-1 expression level was reduced when animals were given febuxostat. We conclude that febuxostat as well as ROS scavengers act on both caspase-1-dependent and -independent pathways of cytokine/chemokine release by CCs. Further studies are required to clarify the underlying mechanisms of the involvement of XO in caspase-1-dependent and -independent pathways.

We hypothesize that increased XO activity eventually causes excess ROS formation, leading to tissue damage. In this context, pharmacological inhibitors of XO, such as febuxostat, allopurinol and oxypurinol, have been reported to have anti-inflammatory effects in various diseases, most likely via inhibition of ROS production. In addition, we recently demonstrated inhibitory effects of febuxostat on the expression of cytokines/chemokines *in vitro* in human and murine macrophages. This inhibitory effect was related to intracellular ROS levels and not to uric acid levels^11^. On the other hand, uric acid, which is one of the end-products of XOR, can act as scavenger for peroxynitrite (PN). Thus, uric acid in the blood is expected to contribute to the reduction of oxidative stress by PN. Indeed, PN has been implicated in multiple sclerosis (MS) and its animal model experimental allergic encephalomyelitis (EAE), and uric acid has been reported to be reduced in the serum of patients with multiple sclerosis (MS) and optic neuritis (ON). It has been suggested that lower serum uric acid levels in MS patients may represent a loss of protection against PN. In accordance with this, uric acid administration has been beneficial in EAE. However, some recent studies failed to correlate uric acid serum levels and several clinical parameters of MS and ON^30^,^31^. Stroke is also another disease in which uric acid could play a protective role. However, the prognostic value of serum uric acid in acute ischemic stroke is controversial. In one clinical study, increased uric acid serum levels are associated with better outcome in patients with stroke^32^, whereas in another study no association between serum uric acid levels and both short- and long-term outcome in stroke was found^33^. Most importantly, a causal relationship between increase in stroke and decrease in uric acid levels by febuxostat treatment has not been established and no exacerbations of inflammatory diseases were reported in patients with febuxostat^34^,^35^.

Among clinically available XOR inhibitors, allopurinol as well as febuxostat has been reported previously to be effective in mouse model of atherosclerosis^9^. Our recent studies showed that febuxostat attenuated LPS-induced MCP-1 production^11^, and improved endothelial dysfunction in ApoE^−/−^ mice (see Supplementary Fig. S1 oneline). Febuxostat is 1000-fold more potent than allopurinol in the inhibition of ROS or uric acid production by XO, and it can completely inhibit ROS production by endothelium-bound XO whereas allopurinol only has a partial effect^36^. In high-risk cardiac surgery patients with hyperuricemia, febuxostat but not allopurinol lowered oxidized LDL level, oxidative stress and pulse wave velocity^37^. These findings indicate that febuxostat has superior potency to allopurinol for inhibition of XO-derived ROS production. Furthermore, the finding that febuxostat can inhibit the production of caspase-1-dependent and -independent cytokine/chemokine makes it an attractive therapeutic agent in atherosclerosis.

## Methods

### Animal experiments

Male apoE^−/−^ (B6.129P2-*Apoe^tm1Unc^*/J) and wild-type (WT; C57BL/6J) mice were purchased at 7 weeks of age from Jackson Laboratory (Bar Harbor, ME). From 8 weeks of age, apoE^−/−^ mice were fed with western diet containing 0.15% cholesterol and 40% fat (F2WTD; Oriental Yeast, Tokyo, Japan), and administrated with drinking water containing 0.027 mg/mL of febuxostat (Teijin Pharma Ltd., Tokyo, Japan) for 12 weeks. As non-atherosclerotic control, WT mice were kept on normal diet for the same period. All experimental procedures were conducted in accordance with the Guiding Principles for the Care and Use of Laboratory Animals (Teijin Pharma Ltd.), and each experimental protocol was approved by the Committee for Animal Experiments of the Teijin Institute for Biomedical Research. All efforts were made to minimize suffering and minimize the number of mice needed to assess statistical significance and experimental reproducibility.

### Histology and lesion analysis

For immunohistochemistry, aortic root was frozen in O.C.T compound, cut into 10 μm serial section and stained with MOMA-2 antibody (AbD serotec, Raleigh, NC) and XOR antibody (Santa Cruz Biotechnology, Inc., Santa Cruz, CA) for assessment of macrophage infiltration, and for examination of XOR protein level, respectively. For quantification of atherosclerotic lesion, Oil Red O-positive lesion surface areas on *en face* preparation of whole aorta were measured. Briefly, aorta from root to the abdominal area was dissected and fixed with formalin, and followed by removing the connective tissues carefully. Then, the entire aorta was opened longitudinally, pinned *en face*, stained with Oil Red O (Wako, Osaka, Japan), and photographed with digital camera. The total surface area and total Oil Red O-positive lesion area were determined using WinROOF ver.6 (Mitani Corporation, Tokyo, Japan). The extent of atherosclerotic lesion development was defined as the percentage of total Oil Red O-positive lesion area over the total surface area. In addition, frozen sections of aortic root were also stained with Oil Red O, and total Oil Red O-positive lesion area was determined. For detection of superoxide, frozen sections were stained with 10 μM of dihydroetidium (DHE; Wako) for 30 min, followed by counterstaining and sealing with VECTASHIELD® Hard set Mounting Medium with DAPI (Vector laboratories, Burlingame, CA). Fluorescence images were obtained with BIOREVO BZ-9000 apparatus (KEYENCE, Japan), and fluorescence intensity was analyzed with ImageJ software (NIH, Bethesda, MD).

### Isometric tension measurements

Two murine aortic rings (2 mm length) were prepared and set into magnus apparatus (Iwashiya Kishimoto Medical Instruments, Kyoto, Japan) in Krebs-Henseleit solution (119 mM NaCl, 25 mM NaHCO_3_, 10 mM glucose, 4.7 mM KCl, 1.2 mM MgSO_4_7H_2_O, 1.2 mM KH_2_PO_4_, and 2.5 mM CaCl_2_2H_2_O) with 95% O_2_/5% CO_2_. After equilibration for 60 min, rings were contracted twice with 60 mM KCl and once with 1 μM of phenylephrine (PE; Wako). Then, the relaxation reaction was obtained by addition of acetylcholine (ACh; Sigma-Aldrich, St. Louis, MO) to confirm the presence of endothelial cells. After washing, cumulative addition of ACh (0.001–100 μM) to PE-precontracted ring was performed to obtain endothelial cell-dependent relaxation. Cumulative addition of sodium nitroprusside (SNP; 0.001–100 μM; Sigma-Aldrich) to PE-precontracted ring was performed to obtain endothelial cell-independent relaxation.

### Measurement of XO/XDH activity

XO/XDH activity in tissues or plasma was measured with pterin-based assay as previously described^38^. For *in vivo* samples, aorta or liver tissues were homogenized in 50 mM phosphate buffer (pH 7.4) containing 1 mM EDTA and protease inhibitor cocktail. Then, tissue homogenates or plasma were reacted with 50 μM pterin (Sigma-Aldrich) for XO activity, or 50 μM pterin plus 50 μM methylene blue (Sigma-Aldrich) for XO plus XDH activity. Activity was expressed as units/mL (plasma) or units/mg protein (tissue homogenate) using buttermilk XO (Merck Millipore, Billerica, MA) as standard.

### Blood parameters

Blood samples were collected 12 weeks after administration with febuxostat. Plasma concentration of total cholesterol, HDL-cholesterol, triacylglycerol, glucose, and NEFA were measured by enzymatic methods using Hitachi 7180 autoanalyzer (Hitachi, Tokyo, Japan).

### Quantitative real-time RT-PCR (qRT-PCR)

Total RNA from aorta was extracted using TRIzol reagent (Life Technologies, Carlsbad, CA) according to the manufacturer's instructions, and converted to cDNA using SuperScript VILO MasterMix (Life Technologies). The cDNA was amplified using SYBR® Green (Life Technologies) with gene-specific primers on ABI PRISM 7500 system (Life Technologies). The oligonucleotide primers used in the experiments were listed in Table 2. For data normalization, an endogenous control (36B4) was determined for controlling the cDNA input and the relative units were calculated by a comparative *C*t method.

### Preparation of cholesterol crystal

Sterile, pyrogen-free CCs were synthesized as described previously^15^. Prior to experimentation, crystals were resuspended in RPMI1640 medium (Life Technologies), grounded and sonicated for 30 min. As expected from pyrogen-free crystals, these crystals contain no contaminants able to prime cells, and consequently no IL-1β was detected into supernatants of unprimed macrophages stimulated with CCs.

### Bone marrow-derived macrophage preparation

Bone marrow cells were isolated from the tibia and femurs of wild-type C57BL/6 mice. The isolated cells were incubated for 7 days on Petri dishes with 30% L929 conditioned medium (source of M-CSF), 10% FBS (PAA laboratories GmbH, Austria), 1% HEPES (Life Technologies) and 1% penicillin-streptomycin (Life Technologies) in Dulbecco's Modified Eagle Medium (DMEM, Life Technologies) for differentiation into bone marrow-derived macrophages (BMDM). After differentiation, the resulting BMDM were detached using cold PBS, and plated for stimulation experiments with complete RPMI1640 medium with 10% FBS, 1% HEPES and 1% penicillin-streptomycin (Life Technologies).

### Cell stimulation

BMDM were plated and left several hours in 96-well plates or 48-well plates in complete medium. For experiments that required priming, cells were stimulated overnight with 100 ng/mL of Pam3CSK4 (InvivoGen, San Diego, CA). Cells were then incubated in incomplete medium (without FBS) with febuxostat at the indicated concentrations, NAC (50 mM, Sigma-Aldrich), Apocynin (500 μM, Sigma-Aldrich) or z-VAD-fmk (10 μM, ENZO Life Sciences, New York, NY) prior to stimulation with CCs (1 mg/mL) for 6 h. As a positive control, MSU crystals at 250 μg/mL were used. At the end of the incubation, supernatants were collected and stored at −20°C for ELISA. Cell extracts were prepared for XOR activity and uric acid measurement.

### Measurement of LDH

LDH in supernatant was measured using CytoTox-ONE™ Homogeneous Membrane Integrity Assay kit (Promega, Madison, WI) according to the manufacturer's instructions. LDH release (%) was calculated by using the following formula. LDH release (%) = [(value in sample) − (background)]/[(value in Triton X-100-treated sample) − (background)].

### ELISA

MCP-1, IL-1β, IL-6 ELISA kit (eBioscience, Inc., San Diego, CA) and IL-1α ELISA kit (BioLegend, San Diego, CA) were used to measure the corresponding chemokine/cytokines levels in supernatants according to the manufacturer's instructions.

### Measurement of intracellular ROS level

Intracellular ROS level was measured with Cell Meter™ Fluorimetric Intracellular Total ROS activity assay kit (AAT Bioquest®,Inc., Sunnyvale, CA) according to the manufacturer's instructions. Briefly, cells were loaded for 60 min with Amplite™ ROS green. Then, cells were pretreated for 10–15 min with the indicated concentration of febuxostat or NAC, and stimulated with 1 mg/mL CCs in HEPES-buffered saline (Life Technologies). Fluorescence intensity on 490 nm (excitation) and 525 nm (emission) was measured with fluorescence plate reader.

### Measurement of intracellular uric acid

Intracellular uric acid was measured with Amplex® Red Uric Acid/Uricase Assay Kit (Life Technologies) according to the manufacturer's instructions. Uric acid level was expressed as nmol/mg protein by normalizing with protein concentration.

### Statistic analysis

All data are expressed as mean ± SEM. For two-group comparisons, student's *t* test was used. For multiple comparisons, one-way ANOVA followed by a Dunnett's test was used to compare each group versus a vehicle-treated or medium group. All data were statistically analyzed using GraphPad PRISM software version 6.01 (GraphPad, La Jolla, CA). Differences with a probability value of <0.05 were considered significant.

## Author Contributions

J.N., N.B., T.S. and M.T. designed experiments. J.N., C.M., S.T. and T.S. performed experiments. J.N., N.B., A.I. and A.S. wrote or contributed to the writing of the manuscript. T.K., A.S. and Y.Y. supervised this research.

## Supplementary Material

Supplementary Information Supplementary Information

## Figures and Tables

**Table 1:** Comparisons of body weight and plasma parameters in WT, vehicle- or febuxostat-treated ApoE^−/−^ mice

	WT	ApoE^−/−^	ApoE^−/−^
Parameters	Vehicle	Vehicle	Febuxostat
BW (g)	29.3 ± 0.4	36.1 ± 1.6†	37.5 ± 0.9*
T chol (mg/dL)	69.2 ± 2.8	1499.8 ± 46.6*	1406.0 ± 90.8*
HDL (mg/dL)	42.0 ± 2.1	10.8 ± 1.2*	14.4 ± 1.0^§^*
TG (mg/dL)	74.8 ± 9.7	165.8 ± 17.9†	204.6 ± 20.9*
NEFA (mg/dL)	405.6 ± 31.0	698.6 ± 55.6†	681.8 ± 46.4†
Glucose (mg/dL)	202.6 ± 7.2	237.4 ± 9.3‡	246.8 ± 7.5†
XO activity (mU/mL)	62.3 ± 1.8	87.4 ± 1.9*	54.9 ± 3.9||

Plasma was prepared after 12 weeks from vehicle-treated WT mice or from vehicle- or febuxostat-treated ApoE^−/−^ mice. Values are mean ± SEM. BW indicates body weight; T chol, total cholesterol; HDL, high density lipoprotein; TG, triacylglycerol; NEFA, non-esterified fatty acid. XOR activity in plasma was derived from XO, but not XDH. Samples numbers: n = 20 for XO activity and n = 10 for the other parameters.

*P < 0.0001;

^†^*P* < 0.001;

^‡^*P* < 0.01 vs. WT;

^§^*P* < 0.05;

||*P* < 0.0001 vs. vehicle-treated ApoE^−/−^ mice.

**Table 2:** Sequences of primer pairs used in this study

Genes	Forward (5′ → 3′)	Reverse (5′ → 3′)
MCP-1	GGAAAAGGTAGTGGATGCAATTAGC	AACTGCATCTGCCCTAAGGTCT
IL-1α	CGAAGACTACAGTTCTGCCATT	GACGTTTCAGAGGTTCTCAGAG
IL-1β	TCCAGGATGAGGACATGAGCAC	GAACGTCACACACCAGCAGGTTA
eNOS	GAGATCAAAGGGCTACAACCTG	TAGAGATGGTCCAGTTGGGAG
CD68	TCAAACAGGACCTACATCAGAG	GAAGGACACATTGTATTCCACC
VCAM-1	CCCAAACAGAGGCAGAGTGT	CAGGATTTTGGGAGCTGGTA
36B4	GGCCCTGCACTCTCGCTTTC	TGCCAGGACGCGCTTGT
