# Supplementary material for: Xanthine Oxidase Inhibition by Febuxostat Attenuates Experimental Atherosclerosis in Mice
Source: Sci Rep. 2014 Apr 1;4:4554. doi: 10.1038/srep04554 (PMC3971401; doi:10.1038/srep04554)
Supplement: Supplementary Information [file srep04554-s1.pdf]

Xanthine Oxidase Inhibition by Febuxostat Attenuates Experimental Atherosclerosis in Mice

Johji Nomura*[1,2], Nathalie Busso[2], Annette Ives[2], Chieko Matsui[1], Syunsuke Tsujimoto[1], Takashi Shirakura[1], Mizuho Tamura[1], Tsunefumi Kobayashi[1], Alexander So[2], Yoshihiro Yamanaka[1]

[1]Pharmaceutical Department Research Laboratories, Teijin Institute for Bio-Medical Research, Teijin Pharma Limited, Hino, Tokyo, Japan, [2]Service of Rheumatology, Department of l'Appareil Locomoteur, Centre Hospitalier Universitaire Vaudois, University of Lausanne, Lausanne, Switzerland

Supplementary Figure S1

Febuxostat Inhibits Endothelial Dysfunction in ApoE$^{-/-}$ Mice

Increased levels of ROS can react with nitric oxide to generate metabolites that result in endothelial dysfunction[1]. We therefore examined whether the inhibition of ROS generation by febuxostat had an effect on endothelial dysfunction in ApoE$^{-/-}$ mice. ACh-responsiveness of aorta from ApoE$^{-/-}$ mice was significantly reduced compared to that from WT mice, indicating endothelial dysfunction in aorta of ApoE$^{-/-}$ mice. Treatment with febuxostat for 12 weeks significantly improved ACh-responsiveness in ApoE$^{-/-}$ mice. On the other hand, SNP-induced relaxation and PE-induced contraction were not affected by febuxostat treatment. Altogether, these data suggest that the increased XOR and subsequent oxidative stress in endothelial cells, cause endothelial dysfunction.

Supplementary Figure S1. Febuxostat inhibits endothelial dysfunction in ApoE$^{-/-}$ mice. **a,** Endothelial cell-dependent relaxation to ACh in PE-precontracted aortic rings from vehicle-treated WT (n=8), vehicle-treated ApoE$^{-/-}$ (n=9), and febuxostat-treated ApoE$^{-/-}$ mice (n=8). Data are pooled from two independent experiments in which similar results were obtained, and shown as mean±SEM. $^{\#}P<0.05$, $^{\#\#}P<0.01$ versus vehicle-treated WT,

*$P<0.05$, **$P<0.01$ versus vehicle-treated ApoE$^{-/-}$ mice. **b,** Endothelial cell-independent relaxation to SNP in PE-precontracted aortic rings from vehicle-treated WT (n=8), vehicle-treated ApoE$^{-/-}$ (n=9), and febuxostat-treated ApoE$^{-/-}$ mice (n=8). Data are pooled from two independent experiments in which similar results were obtained, and shown as mean±SEM. **c,** Contraction to PE in aortic rings from vehicle-treated WT (n=8), vehicle-treated ApoE$^{-/-}$ (n=9), and febuxostat-treated ApoE$^{-/-}$ mice (n=8). Data are pooled from two independent experiments in which similar results were obtained, and shown as mean±SEM.

1. Mudau, M., Genis, A., Lochner, A. & Strijdom, H. Endothelial dysfunction: the early predictor of atherosclerosis. *Cardiovasc J Afr* **23**, 222-31 (2012).

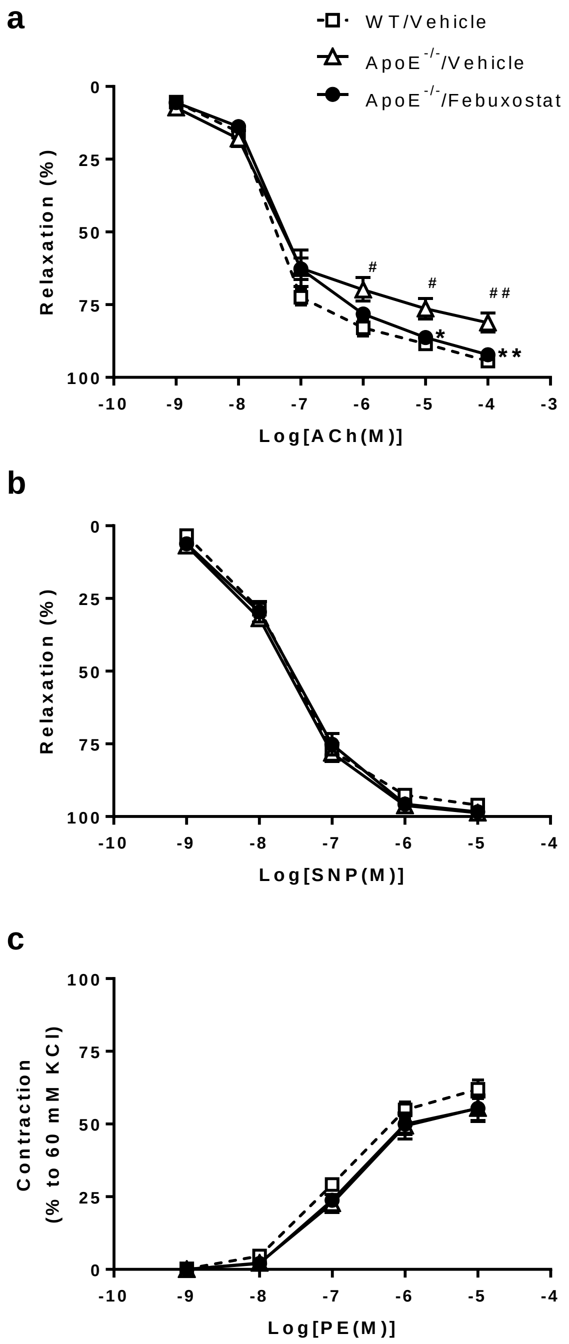
a
WT/Vehicle
ApoE-/-/Vehicle
ApoE-/-/Febuxostat
Relaxation (%)
Log[ACh(M)]
b
Relaxation (%)
Log[SNP(M)]
c
Contraction
(% to 60 mM KCl)
Log[PE(M)]
